# Negotiating cohabitation in a Nigerian abattoir: One Health perspectives of human-animal-ecosystem interactions examined in the light of the SARS-CoV-2 pandemic

**DOI:** 10.1186/s42522-025-00161-9

**Published:** 2025-07-16

**Authors:** Hellena Debelts, Valerie Allendorf, Olayinka Asala, Ebere Roseann Agusi, Ismaila Shittu, Oluyemi Ogunmolawa, Judith Bakam, Bitrus Inuwa, Jeremiah Ijomanta, Joshua Seyi Oyetunde, Chinonyerem Chinyere, Austine Elah, David Oludare Omoniwa, Klaas Dietze, Adeponle Adeoye, Chinwe Lucia Ochu, Anja Globig, Almudena Mari-Saez, Clement Meseko

**Affiliations:** 1 Robert Koch-Institut, Berlin, Germany; 2 Friedrich-Loeffler-Institut, Suedufer 10, 17493 Greifswald-Insel Riems, Germany; 3 National Veterinary Research Institute, Vom, Plateau State Nigeria; 4 Department of Veterinary Services Federal Capital Territory Administration, Abuja, Nigeria; 5 Faculty of Veterinary Medicine, University of Jos, Jos, Plateau State Nigeria; 6 Department of Sociology, Redeemer’s University, Ede, Osun State Nigeria; 7 Nigeria Centre for Disease Control and Prevention, Abuja, FCT Nigeria; 8 TransVIHMI, Université de Montpellier, INSERM, Institut de Recherche pour le Développement Délégation Régionale Occitanie, Montpellier, France

**Keywords:** One Health, Abattoir, Nigeria, Livestock, Anthropology, Urbanization, SARS-CoV-2, Wildlife

## Abstract

**Background:**

Urbanization and intensifying interactions between humans, animals, and the environment present complex challenges for One Health, particularly in regions like Central Nigeria. This study investigates the dynamics of human-animal-environment interfaces within a Nigerian abattoir during the ongoing SARS-CoV-2 pandemic, focusing on zoonotic transmission pathways and multispecies interactions.

**Methods:**

We employed a mixed-methods approach, combining ethnographic observations, semi-structured interviews, and biological sampling of livestock, dogs, and small mammals. Virological analyses included RT-qPCR for SARS-CoV-2 RNA detection and ELISA for antibody identification. Fieldwork spanned rainy and dry seasons in 2022 to capture seasonal variations in human-animal interactions.

**Results:**

The abattoir compound is centre of life of multiple species, involving humans, livestock, synanthropic mammals, and domestic animals, and as such, serves as a dynamic hub for their interactions. Seasonal changes influence interaction intensity, with higher densities during the rainy season. While SARS-CoV-2 RNA was not detected in animal samples, antibodies were identified in cattle, goats, sheep, dogs, and shrews, with higher occurrence in the rainy season. These findings suggest prior exposure of the animals to SARS-CoV-2, underscoring the influence of environmental and human behavioural factors on zoonotic transmission risks.

**Conclusions:**

This One Health study demonstrates the value of integrating ethnographic insights with serological data, and highlights the abattoir compound as a critical site for zoonotic pathogen circulation. Findings emphasize the need for targeted interdisciplinary surveillance and mitigation strategies in high-density, multispecies environments to address zoonotic disease risks and enhance health resilience across species.

**Supplementary Information:**

The online version contains supplementary material available at 10.1186/s42522-025-00161-9

## Background

Living beings, be they human, non-human animals or microbes, are by virtue of sharing the same environment entangled in a sensitively balanced network [1, 2]. Locally, this interconnectedness may be very stable, but it remains vulnerable to the effects of the unusual, or external to the balanced ecosystem [3–5]. Human behavior is a leading driver of disturbance of these equilibria and with that, of disease emergence [6].

Considering biodiversity on one hand and trends in human encroachment in cases such as urbanization and economic growth at the other, Nigeria is one of the predicted global hotspots of disease emergence [7]. With approximately 51.2% of the population already urbanised and a continuing urbanisation trend, especially Nigeria’s capital and the surrounding settlements are facing unprecedented annual growth rates of up to 4% per annum [8]. This rapid urban growth poses challenges related to housing, food security and climate change, affecting livelihoods and the environment [9, 10]. Moreover, population growth and increasingly dense urban and peri-urban centres challenge the traditional ways of supplies with animal products. The steadily rising meat demand and the need for short range of transportation of meat due to restrictions in cold chain availability bring people and livestock closer together. Particularly for urban dwellers of the middle- and lower-income groups, making a significant proportion of Nigerians, livestock is crucial for their livelihoods. Hence, urbanization encompasses the encroachment of humans and livestock alike into former habitats and refuges of wildlife, leading to multispecies urban centres with potential implications for the emergence of zoonotic diseases [11, 12].

Especially in Nigeria, this livestock sector is expanding rapidly to meet the growing demand for animal protein of the growing population (FAO 2019). The meat value chain begins in peri-urban or rural areas with predominantly small-scale livestock farming. It ends with the slaughter and processing of meat products in abattoirs that increasingly become part of these urban and peri-urban landscapes, potentially involving encounters with various humans, other animals, and diverse environmental settings along the way. These abattoirs have and follow different technical as well as hygiene standards, and sometimes function as central knots in animal trade and for animal products through, e.g., integrated market places and holding areas [12]. Research shows that along this chain, contact with animals, animal products, and effluents may facilitate the spillover of animal-derived pathogens to humans [13, 14]. Abattoirs are recognized as potential disease amplifiers and hotspots for a variety of zoonotic pathogens that circulate in human and animal populations, that next to livestock include dogs [15], mosquitos [16, 17], flies [18], that breach into the surrounding residential areas and beyond via human and animal bodies, waste disposal and liquid discharges [19–21]. Other known problems in the industry range from precarious working conditions to the production of unhealthy, exploitative ecologies for humans, animals, and the environment that contribute to the emergence and spread of pathogens [22–26]. This is evident globally, as the meat processing industry has faced repeated multispecies disease outbreaks, including mass outbreaks of COVID-19 among abattoir workers during the pandemic [27–29].

A recent and prominent example for the successful emergence of a novel pathogen is reflected by the COVID-19 pandemic. Through a chain of incidences triggered by anthropogenic activities a novel virus, SARS-CoV-2, emerged, and in the following was able to travel around the globe in a short time [30]. Originating from a yet unidentified animal reservoir, it has retained its zoonotic characteristics. Exposure of wild or domestic animals to the excretions of infected humans, either directly via droplets, aerosols, or indirectly via fomites, may result in successful transmission, depending on the dose of infectious particles and the susceptibility of the species [31]. The frequency of human cases and therefore successful human-to-human transmission has been shown to follow seasonal variations [32]. Similar seasonal variations can be expected in human-to-animal transmission. Also factors such as human and animal activity, variability in immune system function, or viral infectivity and tenacity play a role in the circulation patterns [33]. If transmission is successful, infected animals can show absence to severe clinical symptoms depending on the species, and shedding of the virus varies greatly [31]. In ruminants such as cattle, sheep and goats, SARS-CoV-2 replication seems to be restricted to the upper respiratory tract, where its RNA can be detected only for one to four days post infection in low copy-numbers [34–37]. Seroconversion can be observed from seven to ten days post infection. In healthy dogs, SARS-CoV-2 infection may cause mild signs of respiratory or gastrointestinal disease [38]. Two to four days post infection, viral RNA can be found in nasal swabs. Antibodies can be detected from 10 to 14 days post infection [39], but titres may fade out similarly to those of cats after three to four months [40]. Synanthropic small mammals such as brown rats in urban centres were shown to be infected and seroconverted [41].

Similar to many other countries around the world, Nigeria reported numerous waves of COVID-19, each characterized by the emergence of a novel variant. The abundance of SARS-CoV-2 within the human population in Nigeria, using the daily case reports published by the Nigerian Centre for Disease Control (NCDC) as a proxy, was considered comparatively low throughout the pandemic, with a maximum of 8.9 cases per million inhabitants reported on 27 December 2021 [42]. An analysis and extrapolation of the Nigerian case numbers by Adebowale [43] predicted seasonal variations with an increase of cases in the first and the third quarters of the year. Geographically, the highest case numbers were recorded in the states encompassing the urban centres of the country like Lagos and FCT [44]. Interestingly, a seroprevalence survey conducted in July/ August 2021 during the third wave of SARS-CoV-2, dominated by the Delta variant [45], found that 75.8% of the population in the Federal Capital Territory (FCT) in central Nigeria had SARS-CoV-2 specific antibodies at that time, indicating a much higher infection rate (Kolawole et al. 2022). According to the National Primary Health Care Development Agency, 16% of the eligible population had received their first vaccination dose and 7.6% their second by end of February 2022 [46, 47].

In this study, we aimed to utilise the ability of SARS-CoV-2 to cross inter-species boundaries to investigate the transmission pathways and the likelihood of zoonotic transmissions of respiratory viruses in multiple direct and indirect interfaces among various species cohabiting in a complex shared environment of an abattoir in central Nigeria, using animal samples as indicator for anthropo-zoonotic transmission and by describing the activities and drivers of expositions of beings within the multispecies meshwork of the abattoir during an ongoing pandemic. While epidemiological and statistical methods are often used within the One Health framework to calculate interaction probabilities among humans, animals, and the environment, they struggle to capture the everyday complexities of these interconnections [48]. To address this, we integrated a mixed method approach of anthropological, virological, and empirical methods to examine a specific abattoir ecosystem, emphasizing the multi-species and environmental entanglements central to contemporary One Health concepts [49]. Biological sampling in the abattoir was limited to non-human species. Thus, the social science component of the study was adapted to strongly incorporate human and environmental dimensions, thereby addressing again the key elements of the One Health triad perspective.

## Methods

The research was conducted within an interdisciplinary consortium, consisting of members Nigerian and German federal public health and animal health institutions. The scientific backgrounds of the researchers directly involved were anthropology, biology, human medicine, public health, sociology, veterinary medicine and virology.

Prior to the start of the research, a joint study protocol was drafted by researchers of all disciplines, outlining each discipline’s contributions, their dependencies and their triangulations. This draft of the joint study protocol was discussed, tested and agreed upon at a kick-off workshop in Nigeria. It included the site selection being in FCT as well as the timeline for fieldwork and analysis in rain and dry season, being the two major seasons in Nigeria, to collect data on seasonal differences. From then on, the interdisciplinary dialogue was continued by monthly or bi-weekly virtual meetings on detailed planning and preparations prior to the field work phases, updates during the field work and presenting and extensive discussion of results after the field work phases in virtual or in-person format. Core of the interdisciplinary exchange were the fieldwork periods, in which the anthropology and veterinary team worked for ten days side by side, fostering a direct and immediate exchange and adaptation of the protocol to the actual situation found in the field. The virtual meetings were complemented by annual in-person workshops in Nigeria or Germany for intensive work on data triangulation and preparation of publications.

The site of the study was the compound of a livestock market and abattoir for large and small ruminants located in a satellite town of the metropole area of the FCT, Nigeria. The facility is embedded in a dense, multi-ethnic and rapidly growing settlement with further critical infrastructure, such as a primary health care centre, schools, markets, churches and mosques. Ethical approval was granted by the National Health Research Ethics Committee of Nigeria (Permission No. FHREC/2022/01/08/04-02-22) and by the Animal Ethics Committee at National Veterinary Research Institute (Permission No. AEC/03/118/22). Access to the study site was achieved by visits to the management level by representatives of the project partners. Participation in the study was always voluntary and only possible after signing a prepared informed consent form. The ethical approval did not extend to the collection of samples of humans within our study.

### Anthropological fieldwork

The anthropological component of this study, serving as the community entry point and laying the groundwork for the other two study elements, employed ethnographic methodologies. Daily participant observation, combined with informal discussions and conversations, spanned the twelve-week study period, six weeks in rain and dry season respectively in 2022. Additionally, twenty semi-structured key-informant interviews were conducted in English or in Pidgin English (Questionnaires are available in supplementary file 5). Participants for interviews were selected purposefully to ensure representation across various groups, including professional, religious and ethnic backgrounds. As the majority of people working in the abattoir are men, this is also represented in the interviews. Fieldnotes from observations, photos, and interview transcripts were continuously reviewed and discussed with the veterinary team in the field to identify emerging insights and recurring patterns in answers and observations. The data was then organized and analysed based on pre-selected codes, covering aspects such as transmission routes, interactions between species and the environment, COVID-19, health-seeking knowledge and behaviour, perception of zoonotic risk, and more. Immediate engagement with the emerging data led to the pursuit of new relevant areas of interest, which were then organized along new (sub-)codes.

### Veterinary sampling

Following the anthropologist’s findings on the human-animal encounters and interactions, convenience samples were taken by the veterinarians from slaughtered livestock on seven consecutive days in each season; small mammals were trapped on four consecutive nights within the premises of the abattoir area. Other domestic animals encountered within the premises were sampled as available.

Nasopharyngeal and blood samples from cattle were collected at slaughter, mandibular lymph node and lung tissues taken during evisceration. Therewith, data on breed and sex of each individual were gathered (Supplementary file 1). From each slaughtered small ruminant, a nasopharyngeal swab, blood and the mandibular lymph node were obtained. From dogs encountered on site, oropharyngeal swabs and blood samples were obtained, if possible.

Small mammals were trapped using Sherman live traps (H.B. Sherman Traps Inc., Tallahassee, FL, USA) baited with smoked fish and fried bean cake. Traps were set in places identified as passages for rodents, and in places with reported high rodent activity. The trapping rate was calculated as the proportion of catches of the same species to the overall trapping nights in each season. All trapped small mammals were transported to a temporary field laboratory offside the abattoir premises, where they were handled under the use of adequate personal protective equipment. They were euthanized by halothane inhalation followed by cervical dislocation. Consecutively, body measurements for phenotypic species determination were taken (Supplementary file 3) and thereafter necropsy was performed. Blood was obtained by puncture of the left ventricle, and heart, liver, spleen, lung, and one kidney were collected and pooled in one tube; intestinal sections were collected in a separate tube. Blood samples were left for coagulation and serum was separated. All collected samples were stored in liquid nitrogen until the arrival at the Biosafety-Level 3 laboratory at the NVRI in Vom, Plateau State, Nigeria where they were transferred to a -80^O^C freezer until further analysis.

### Laboratory analysis

Frozen tissue samples were homogenized in 1 ml of Minimum essential medium (MEM) containing antibiotics using Tissue Homogenizer (MiniBeadBeater-16, Biospec, USA). From pooled oropharyngeal or nasopharyngeal swabs and from pools of the clarified supernatant of homogenized tissues, total RNA was extracted using Qiagen RNA mini prep-kit (Qiagen Hilden, Germany) according to manufacturer’s protocol. The extracted RNAs were subjected to Sarbecovirus specific RT qPCR targeting the envelope (E) gene described by Corman et al. [50], positive RNAs were further tested for a SARS-CoV-2 specific RT-qPCR (IP4) [51].

For a subset of small mammals caught, species identification was performed by cytochrome B analysis as published by Schlegel et al. [52] of DNA extracted from the organ pool.

Sera extracted from the blood samples were heat-treated at 56 °C in a water bath (XMTB Baths, HH-W40, China) for 60 min for complement inactivation and decontamination. They were screened with an Enzyme Linked Immunosorbent Assay (ELISA) based on the receptor binding domain (RBD) of different variants of SARS-CoV-2, as described by Wernike et al. [53].

## Results

At the research site, the abattoir is an integral part of the settlement’s composition. People come to buy or sell animals, meat and other food products, or services. Human and non-human beings interact throughout day and night. In such a place, numerous infectious agents are likely to be present and to circulate in close proximity, engaging in dynamic exchange with the surrounding settlement via multi-species bodies and other modes of movement. Due to the large number of actors and different spatial characteristics, we structured the results into subsections, separated by the main driver and central character of the activities observed and described. The main multi-species characters identified in the study are namely humans, ruminants, dogs, rodents and shrews, and SARS-CoV-2.

### The abattoir

**Fig. 1:**
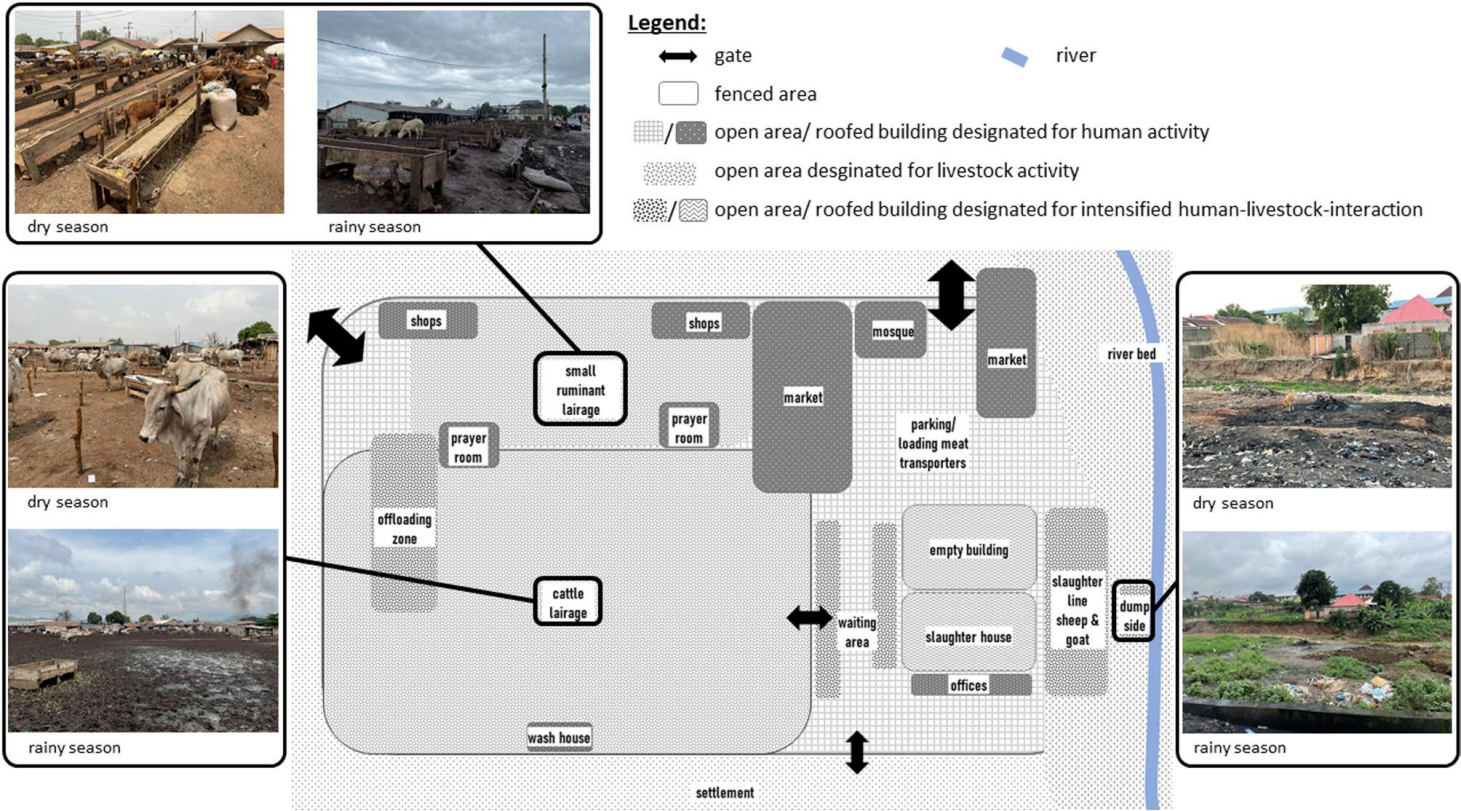
Outline of the compound of the livestock market and the adjacent abattoir. Main structures and buildings are labelled according to their intended usage, and are colour-coded according to the species most active and prevalent within the designated utilization. For the three areas of the cattle lairage, the small ruminant lairage and the river bed/ dump site, the seasonal differences are depicted with photos taken by the research team

Managed by the Ministry of Agriculture under the Abuja Municipal Area Council (AMAC) through a stationed manager, the abattoir supplies Halal meat to the entire FCT and customers in neighbouring states. It operates every day of the year. The compound is fenced in on three sides and borders openly on a small river. The brick walls enclose a large open-air cattle market and a partly roofed, partly open-air goat and sheep market. The abattoir premises also comprise a roofed and paved marketplace with butchers’ tables for meat products and shops for vegetables, some other groceries and general services. Additionally, there are two prayer rooms and a mosque situated within the abattoir area (Fig. 1).

The main slaughter site is located in a slaughterhouse where cattle and occasionally dromedaries are slaughtered and butchered in a large hall, while sheep are slaughtered in a separate room aside of the hall. Goats are mainly slaughtered outdoors on a paved platform facing the river bed. Next to this, the goat carcasses are singed over small chimneys, washed in water basins and eviscerated. Around 100 to 150 cattle and 300 to 450 small ruminants are slaughtered daily, depending on the weekday and season. Around 1000 cattle and a similar number of small ruminants are staying in the respective lairages variating again, sometimes greatly, by weekday and season. Quality control is carried out by stationary veterinarians and animal health workers in ante- and post-mortem inspection of the slaughtered animals. Both professions are employed by the abattoir management. The same applies for the *Mallams*, a regional term for men educated in Koranic studies who are authorized to conduct the Halal slaughter.

After the daily main slaughtering activities, the abattoir and its surroundings are washed down with water by cleaners hired by a cleaning company. The rest of the hundreds of people working at the abattoir are either registered and organised in a union, work for a person who is part of such a union, or come on a daily basis to offer their labour. The majority of ethnic backgrounds in the abattoir are Hausa and Fulani, however, it is a place attracting many people from various groups for work and business. The main religious background of the people working in the abattoir is Islam.

### Humans’ activities at the abattoir

#### Day time

Apart from the veterinarians and health inspectors of the abattoir management, abattoir workers follow different tasks. About fifty butchers are registered in the butchers’ union. Most of these butchers have up to ten assisting personnel that help in daily abattoir work - from animal caretakers, animal traders, butchers’ assistants in the slaughterhouse, meat sellers to meat or animal suppliers, vehicle drivers and water carriers. The majority of the workers come from states in Northern Nigeria.

Work in the abattoir, at the *slaughter slab* (slaughtering line), the butcher tables, the dehairing and evisceration area and also in the livestock lairage commences as early as 5 am and precedes until most slaughtering is done around noon. The market activities continue until the evening. Trucks carrying livestock cargo that either arrive from distant Northern markets or are prepared for to smaller surrounding or distant Southern markets. Throughout the morning and afternoon, customers arrive to purchase animal products and livestock, and engage in various trading and service activities.

Throughout the day, the temporary owners of the livestock on display at the livestock markets attend to their animals’ needs, including feeding, watering, and occasional washing, while waiting for customers. After often thoroughly examining the animals, and once customers and owners agree upon a price, the animals are taken to the slaughter slab, inside or outside the slaughterhouse. There they are slaughtered and processed by the butcher teams on the cemented floor, resulting in various products, such as meat and innards, as well as by-products like blood, bile, horns or hoof capsules. The products are either given to the buyer, offered directly at the market or transported to supermarkets, restaurants or hotels in the whole metropolitan area. All liquid waste from slaughtering, cleaning, washing and other processes is diverted into the adjacent river to be swept away by its current. During the rainy season, the creek widens and recovers more of its bed and the products left there from the abattoir.

Concurrently to the livestock market and the slaughtering activity, a range of services and products are offered on the abattoir premises, including food, clothing, nail-cutting, car-washing, utility services, and catering. At any time of the day, until late at night, prepared foods– such as rice or noodles with sauce, grilled meat (*suya*), or stew, all made with meat from the abattoir– are available from stationary shops and occasional setups during breakfast, lunch and dinner times.

#### Evening and night time

In the evenings, abattoir workers as well as other community members gather to dine at these market shops. They find shelter on benches under sheds or within their shops to rest, sleep or watch over the animals around the slaughterhouse, the market area or in the lairage throughout the night.

People use to sleep here but not in all the departments. Like me now, it is not compulsory that I must sleep here but I have to have somebody that is sleeping here. Like in the night, some bring cows in the night. […] Some people use to offload at night. And cows use to fight in the night. Somebody must be there, yes. […] And some people, because of the condition of their villages, no employment, no steady place, they would just come and they will hang around anywhere they see themselves.

Interview, animal dealer, dry season.

At night, public spaces of the abattoir become devoid of human activities but not human presence. Traders sleep close to their animals, to guard them during the night and butchers sleep around the abattoir, as work starts early and the way to their villages is often too far to commute.

#### Porosity of the abattoir space

That animal they kill here, cow, sometimes camel, sometimes they bring camel here, then the ram, sheep, goat, and the goat itself that is an animal that is common in abattoir here. Actually, you know I have goat and some all these chickens, and ducks, these native ones at my home.

Interview, butcher, dry season.

Yes, in addition to the regular ones we have in the abattoir, there are times where I come in contact with, as a vet I do some private practice, so yes I come into contact with companion animals, dogs, cats, poultry.

Interview, veterinarian, rainy season.

Beyond the confines of the abattoir, humans frequently interact with animals in various ways. Veterinarians actively participate in routine vaccination campaigns organised by the Ministry of Agriculture, or engage in a private practice and treat animals in private homes, exposing them to a diverse range of animals in different contexts outside their work in the abattoir. And many people keep a variety of animals at home for extra income or a financial security.

#### Common human health matters and health seeking behaviours

It is important to note that while occupational safety measures were officially recommended and required for the various working groups in the abattoir, their consistent enforcement proved unfeasible at the management level. Workers directly employed by the abattoir management, such as veterinarians, Mallams, and animal health inspectors were provided with coveralls and personal protective equipment (PPE). However, other self-employed work groups inside and outside of the slaughterhouse were responsible for procuring their own protective gear. As a result, the majority of workers either had limited equipment, such as boots, or none at all, with many relying on slippers and daily clothing while performing their tasks. Nevertheless, the butchers’ union implemented a system to support its members during severe illnesses resulting in inability to work. Union members contributed a fixed amount weekly or monthly to a shared fund, which was used to provide financial assistance for various forms of medications, hospital bills, transportation to their villages to receive family care, or other health-related expenses during times of need.

When asked about common health issues, people reported experiencing symptoms such as fever, cough, nausea, diarrhoea, weakness, colds, joint pain, and others on a regular basis. They attribute their symptoms to well-known prevalent diseases like malaria, typhoid, pneumonia, and parasitic infestations, prompting diverse responses. Some individuals consult the neighbouring Primary Health Care Facility (PHC) for rapid testing for malaria, typhoid, or intestinal parasites and seek treatment with the prescribed conventional medicine. However, none of the interviewed study participants went for COVID-19 rapid testing, or knew someone in the abattoir who did, even though it was offered in the neighbouring PHC in the second half of 2022. Others primarily visit nearby pharmacies to obtain medication for their symptoms. Additionally, herbal medicine purchased from vendors at the abattoir, known healers nearby or in their villages or homemade herbal medicines made from roots, leaves, herbs, and other resources are an integral part of disease treatment or health maintenance, as the following quote underlines:

Sometimes, I use traditional medicine. I practise traditional medicine. If I take tablets, they sometimes don’t work on my body, so I turn to traditional remedies. When I have a fever, for example, before taking pharmaceutical tablets, I first use traditional medicine. If the fever doesn’t go away completely with the traditional remedy, I’ll then take tablets to finish the treatment. I make sure to complete the process properly, and afterwards, I usually don’t have any more trouble with the illness.

Interview, butcher, rainy season.

(translation from Pidgin English to English see Supplementary file 4)

The fact that some diseases can be transmitted from animals to humans and vice versa was of common knowledge before COVID but was usually associated with the intense contact during slaughter, as narrated by one of the persons in the abattoir:

You know at time cow use to enter different bush, night and day. Some of them use to carry spirit in their body. There are good and bad spirits and you inhale them. […] So, you are killing them every day and can breathe in the spirits, that thing can affect you at time. So that is what is happening mostly.

Interview, animal dealer, rainy season.

As the last quote illustrates, cattle are considered as bridges between the bush and the abattoir, carrying potential hazardous or congenial entities from the wilderness to the peri-urban community. When being slaughtered, the butchers can inhale these “spirits” and get sick, or– in case of encountering good spirits– health or other beneficial traits such as courage may be transmitted. These explanations for the observed health issues of certain work groups in the abattoir, such as the butchers, follow different lines of reasoning beyond pathogens as causative factors, but are centred around breathing and, in other cases fluids like blood or bile, as a way of exchange and transmission.

#### COVID-19 - impact and perception

According to the majority of the people associated with the abattoir, “Corona”, as COVID-19 was commonly referred to, did not affect anyone’s health at all or in a way that justified the restrictions on their lives, that were in place during 2020 and partly into 2021. These restrictions limited and still limit the number of animals slaughtered and people’s movement and presence in the abattoir, as one of the measures introduced was to reduce the number of people occupying the abattoir space at the same time. Customers were less. People had less or no income. As a practicing Mallam and a previous animal seller, who lost his business during the pandemic restrictions in the abattoir, note:

Before Covid pandemic they would slaughter up to 50 cows at one time in the slaughterhouse. But governmental hygiene restrictions now allow only 25 cows and a cleaning afterwards.

Interview notes with a Mallam, dry season.

During Covid he lost too much and now he is working as an assistant butcher. The other interlocutor explains how he was lucky but still people bought less. Now the prices are also very high. Before Covid, his average cow would be 300.000 Naira, now it is double that price. He continues and says that he does not know of anyone who got sick of Covid in the abattoir: ‘It did not affect our health but our pockets.’.

Interview notes with a butcher and animal dealer, dry season.

Some people had lost their livelihoods and, at the time of the study, were still trying to make ends meet by offering their manual labour at the gate to anyone who came to the abattoir in need of assistance. The pandemic also had a direct impact on economy as a whole, leading to higher prices also for animals and feed. One animal dealer explains how in his perception COVID-19 is not there.

It is a lie. It is a disease for countries like China, in Europe and America but not for me. Not for Nigeria. I do not see it - not like I see malaria, typhoid, tuberculosis around here. It made the government beg and begging is not good. It made business really hard during lockdown. Only two market days were allowed then. I know no one who had Covid or know anyone who knows anyone who had it.

Interview notes with an animal dealer, dry season.

The sampling and testing of animals for the presence of SARS-CoV-2 or on prior exposure to it raised curiosity and interest, but at the same time doubt that it could be found, as it was not experienced and seen by people in daily abattoir life in changes of animal or human health. As people perceived that they were no changes in their health status since the declaration of COVID-19 as a new disease, they did not think their animals could be affected.

### Ruminants’ activities at the abattoir

#### The lairage

At times we buy female ones, at times we buy bigger size, at time we buy small size. At times, like the dry season, we buy the one we can graze, that means to feed them, to keep them aside and feed them […] So I give them steady food day and night and water also steady day and night [in the lairage of the abattoir]. When I do it for 60 days I get some profit. I do it two times in the dry season.

Interview, animal dealer, rainy season.

Yes, we do for fattening and some of them we just decided to, you know, keep them. Just I feel like keeping this one. We just keep them [in the lairage] and we keep some for fattening. […] So that is why in the process you see some of them giving birth here [in the lairage]. […] I keep them like three, four months or five months, the highest six months here.

Interview, animal dealer, rainy season.

Cattle, goats and sheep on the livestock markets of the abattoir come from different parts of Nigeria, mainly from neighbouring and Northern federal states, as well as from neighbouring countries like Niger, Cameroon and Chad. After their arrival, predominantly in the early morning hours, some are slaughtered immediately while others are displayed for the first half of the day to potential customers from inside or outside, who come to the market to select animals either for direct slaughter or to buy live animals to take them to other places, e.g., to the South of the country. At times, herds of small ruminants are transported to Lagos for shipment to the rest of the world. While the trade extends geographically wide in dry season it reduces to more accessible and surrounding areas in rainy season, as a result of flooding and general road conditions. Moreover, most animals stay for shorter periods of time in the lairage in the peak of rainy season, as the environment is less conducive for their health.

Cattle arriving in extremely weak conditions, e.g. unable to stand, are often selected for immediate slaughter either directly in the arrival zone or in the slaughterhouse, to where they are taken in wheelbarrows. Healthy animals remain on the market premises until they are sold. In particular cattle bought undernourished at markets in the crisis-torn Northern Nigeria at the end of the prolonged dry seasons may stay for weeks or months to be fattened during the first part of rainy season for a higher sale price at the abattoir. Some may even stay for several years if they were born in the lairage from cows bought in pregnancy. These farmed animals engage in intense interaction with others, encountering humans and other species in the lairage and as they roam daily through the settlement and adjacent pastures.

#### Seasonality and human-cattle arrangements

.

During the dry season, the majority of the animals stay under clear sky, scattered all over the area of the livestock market (Fig. 1). In the afternoons, animal dealers often send their animals for daily grazing activities to nearby “bushes” at the side of the river, reducing the cost for water and feed. At sunset, all ruminants are directed by their herders to the lots in the lairage belonging to their temporary owners.

During the rainy season, the lairage area is flooded and muddy; the few dry patches under roofs or on small hills are densely populated by waiting livestock (Fig. 1). The temporary yards and sheds in the lairage, where in dry season livestock owners and their workers wait for customers under canopies out of the sun, have been removed.

#### Common bovine health issues

Depending on the state of health of the animals, the temporary owners decide on their fate and further treatment, including the administration of various medicines such as vitamins, appetizers, herbal medicines, and a range of antibiotics available from local para-pharmaceutical shops. The medications are either given or injected to the animals by their owners or the para-pharmaceutical sellers. Only in rare cases the abattoir veterinarians are consulted. There is no system in place to monitor compliance with potentially applicable withdrawal periods.

According to the local veterinarians, the most common infectious diseases in ruminants slaughtered at the abattoir, include bovine tuberculosis, contagious bovine or caprine pleuropneumonia, lumpy skin disease, peste des petits ruminants, and various parasites (Adamu et al. 2021). Depending on the picture presented to the veterinarians during the post-mortem inspection, they can confiscate individual organs or entire animal carcasses. Once the daily slaughtering operations have been completed, these confiscated carcasses or parts of carcasses are brought to the dump site close to the river, where they are burnt together with carcasses of animals that died during transport or due to other reasons but slaughter.

#### SARS-CoV-2 in ruminants

A total of 147 ruminants were sampled in dry season respectively 241 in rainy season. Sampling effort and success differed among the two seasons due to public holidays. The results from the laboratory analysis for SARS-CoV-2 infections in animals for both seasons can be found in Table 1.

In none of the swab or tissue samples, SARS-CoV-2 specific RNA was detected conclusively (cq-value < 35). Of note, the oropharyngeal swabs of two goats sampled in rainy season showed repeated questionable reactions in the IP4 qPCR (cq-values 35–38). The serological results revealed antibody presence in few cattle and goats in dry season (3.5% and 2.3% respectively), in rainy season they were detectable in all three species tested in frequencies ranging from 15.8% in sheep to 34.0% in goats.

**Table 1:** Laboratory results of ruminants sampled

season	species	individuals sampled	swabs positive in IP-4 qPCR	tissue positive in IP-4 qPCR	sera* positive in ELISA absolute numbers and percentage [Clopper-Pearson 95% CI in brackets]	
dry	cattle	57	0	0	2/57	3.5% [0.4–12.1]
	goat	94	0	0	2/86	2.3% [0.3–8.2]
	sheep	5	0	0	0	0.0%
rainy	cattle	120	0	0	22/119	18.5% [12.0–26.6]
	goat	102	0	0	34/100	34.0% [24.8–44.2]
	sheep	19	0	0	3	15.8%

*serum could not be retrieved from all animals

Numbers of individual ruminants sampled and tested positive in PCR for SARS-CoV-2 specific RNA, and in ELISA for SARS-CoV-2 specific antibodies per season and species

### Dogs’ activities at the abattoir

“Some people use to keep dog here. […] some people use to go to hunt with them in dry season. So those ones use to keep the dogs because of hunting.

Interview, butcher, dry season

Yes, dogs because we use to secure our environment [the abattoir] with dogs. We use them in the night, like around 10 pm. So, the dogs will come and roam around.

Interview, market seller, rainy season

Okay, so for the dogs that roam around here, most times they are usually around here [the abattoir and surrounding settlement]. It is only few owners that take their dogs home with them. And then a certain number of them you find them around in the uncompleted buildings, around here.

Interview, veterinarian, rainy season.

Most of the dogs in the abattoir are owned by butchers. They are fed and cared for in various ways by their individual owners and trained to serve as hunting dogs. During the slaughtering process in the morning hours, the dogs roam freely around storage sheds, marketplaces, farmed ruminants and slaughtering sites as part of the abattoir’s multi-species assemblage. Few butchers take their dogs home after work. In the afternoons of the dry season, the dogs often venture out into the “bush” with their owners, the butchers, to participate in pack hunts for potential prey such as monitor lizards, rats, squirrels, civet cats or primates. The prey is chased until it can be caught and killed, with bites and scratches from struggling prey being inevitable. The precious catch– if not for personal consumption - is often sold on the way back to the settlements, as demand for bushmeat is high. If the catch is alive, it may be taken to designated markets for sale.

During the rainy season, hunting activities are suspended, so the dogs roam freely around the abattoir at all times, encountering humans, other dogs and other animals on their way through the surrounding settlement. Direct contact with humans is usually limited to their owners and is generally friendly in nature. When moving around in a pack, their interactions with each other appear to follow a well-established pack dynamic. On their search for food such as leftovers from slaughter and market activities in their abattoir habitat and beyond, the dogs also make use of what they find in the stream bed where the discarded dead animals, body parts and organs are collected to be burned at the end of each workday in the slaughterhouse. The staff responsible for the daily incineration process lament the repetitive task of retrieving scattered fresh or half-burned body parts, which are frequently dispersed by dogs scavenging and consuming them. In the evening, when everything and everyone come to rest, they lie relaxed together with a free-roaming flock of goats and cattle, bathing in the setting sun. At night, they take on the role of guarding the premises, using their sense of smell to distinguish between those who belong to the abattoir and those who do not.

#### SARS-CoV-2 infections

Dogs could only be sampled in rainy season, as they were mostly absent in dry season and therefore not available for sampling. From a total of seven hunting dogs owned by four butchers, blood was retrieved. Oropharyngeal swab samples could not be taken. The serum samples were analysed by ELISA for post-exposure antibodies (Table 2). The sera of four of the seven dogs reacted positive in the ELISA, pointing towards previous exposure towards SARS-CoV-2.

**Table 2:** Laboratory results of dogs sampled

season	species	individuals sampled	swabs positive in qPCR	sera positive in ELISA
rainy	dog	7	no swabs taken	4/7

Number of individual dogs sampled and tested positive in PCR and ELISA for SARS-CoV-2 RNA respectively antibodies. Of note: dogs were only sampled in rainy season, as they were mostly absent in dry season and therefore not available for sampling.

### Rodents’ and shrews’ activities in the abattoir

Of course, many rats. They bother me at night in the quarter [in the abattoir]. Because I experience it in the quarter. Normally I see it.

Interview, cleaner, rainy season.

In the abattoir, “rats” - a collective term for various species of rodents and shrews in the local context of the abattoir - are permanent cohabitants, thriving on the rich supply of all kinds of animal protein and food leftovers. They emerge in the evening hours, to explore their territories. During twilight, they are easily visible roaming through the different drainages around the slaughterhouse or in the market areas. They infiltrate the night quarters of livestock and humans alike, not unnoticed, but barely evitable. People report hearing their sounds, seeing their shadows, or feeling their movements around them or on them, as they occasionally jump on sleeping people. Due to this abundance, “rats” are trapped by some people and killed to control their infiltration, whereas other few people hunt some of the species present at the abattoir for food. During the day, the small mammals reside in the cracks and dark corners of the buildings or in burrows in the open ground along the riverbed. Others come from the settlement, through openings in the outer walls, to roam in search for food.

In dry season, due to the scarce availability of water, thorough cleaning is carried out mainly in the slaughterhouse, while other areas, such as market floors, tables, and the lairage area, are kept tidy but not extensively cleaned with water or detergents. During rainy season, in many areas where slaughtering labour is performed or where organic materials are present, maggots are crawling on the open soil, plastic sheets, in buckets, or in the mud.

Trapping activities within the abattoir premises led to 74 trapping nights in dry season and 82 in rainy season, respectively. Trapping rates for both seasons is shown in Table 3. Cytochrome B analysis determined that only two species were trapped: (i) the shrew *Crocidura fulvastra*, belonging to the *Crocidura olivieri* complex [54] and (ii) the brown rat *Rattus norvegicus* as the only rodent species with a trapping rate of 2.56%. Laboratory results of the small mammals are summarized in Table 3.

**Table 3:** Catching data and laboratory results of small mammals

season	species	trapping rate	individuals caught	organs positive in qPCR	swabs positive in qPCR	sera* positive in ELISA
dry	*Rattus norvegicus*	2.7%	2	0	0	0/1
	*Crocidura fulvastra*	20.2%	15	0	0	3/6
rainy	*Rattus norvegicus*	2.4%	2	0	0	1/2
	*Crocidura fulvastra*	17.1%	14	0	0	0/6

*serum could not be retrieved from all animals

Trapping rates and numbers of individual small mammals sampled and tested positive in for current infection with (qPCR) or post-exposure to (ELISA) SARS-CoV-2, per season and species. Species identification was determined by Cytochrome B analysis

## Discussion

The COVID-19 pandemic unfolds as a multi-species story [55]. It challenges conventional distinctions between the human-made and the natural, as well as the biological and the social [56] to bring processes of intertwined becoming to the fore [57]. Our collaborative interdisciplinary research delved deeply into the diverse facets of a time in the pandemic at the local level, narrating one chapter of this multi-species story from various angles shifting away from the commonly prevalent anthropocentric perspective. This exploration takes place in a setting that is probably not unusual, aiming to highlight complexities of the health landscape of the 21st century [58].

In the abattoir, workers come into contact with a significant number of different fluids such as blood and lymph, when they perform tasks like bleeding, evisceration, and cutting the meat. But as the abattoir is much more than only a place designated to the slaughtering of livestock, it is a hub of contact between humans, livestock, wildlife and pathogens. This contact includes not only direct physical touch of bodies or fluids, but the airborne exchange of breaths by all species, evaporation of fluids and left behind traces on materials and the air through urine, faeces and other excreta.

### Daily activity rhythms and multispecies ecologies in the abattoir

The ecosystem of the abattoir as a biological community follows a well-established daily rhythm of different waves of activity, driven by different species, their different needs and the seasons. The days are busy visibly with human-driven activities. In various roles such as veterinarians, managers, vendors, animal owners, butchers, or cleaners, humans are in constant direct or indirect contact with the many domestic and synanthropic animals in their environment. This exchange can be in form of direct contact, through slaughter or care processes, in the case of touching, caressing, giving injections, killing and cutting meat, selecting meat for buying, disposing carcass parts, cleaning, and more. It can also be indirect, involving remnants on used materials, urine, faeces, saliva on hands and food, or aerosols of exchanged breath and other evaporated particles in the air. Throughout this daily rhythm, livestock follow the rules of their human owners in terms of movement and feeding possibilities. The dogs, however, unless led on hunting trips by their trainers, pursue their own interests, be it food, mates or simple curiosity, keeping their distance from the busiest places.

During the night, other non-human life enters perception of the remaining people. Nocturnal animals such as rats and shrews become more visibly active. These animals invade public spaces, now devoid of human activities but not human presence. In search of sustenance, potential mates, or suitable spaces for new burrows, they traverse every nook and cranny of human structures. Occasionally, they encounter sleeping or resting humans along their way. Similarly, at night the dogs’ behaviour changes from a relaxed to an active and alert state, hunting for small mammals or guarding the area.

The patterns of these daily and nightly encounters in the shared space are strongly influenced by the seasonal variations. During rainy season, life is lived and shared more densely by humans and other animals in smaller areas that remain dry under the cover of wooden sheds, cemented floors and the few concrete but porous building structures. This entanglement of humans, ruminants, dogs, and wildlife and its changing intensities is reflected in the laboratory findings. Even though SARS-CoV-2 specific RNA was not conclusively detected in any of the samples, the ELISA results provide evidence that the animals investigated had been previously exposed to a Sarbecovirus like SARS-CoV-2, and that the exposure increased during the rainy season. The differences in seropositivity between the two seasons were significant for cattle (Chi-square test, *p* = 0.013) and goats (Chi-square test, *p* = 1.4*10^− 7)^. In cats, antibody titres are known to be measurable for four up to six months after exposure [40, 59]. In the absence of data on SARS-CoV-2 antibody persistence in other animals, we assume a similar time period, so that our results from the late rainy season in September 2022 indicate exposure to the virus at some point during the previous six months.

The sampling periods coincided with two preceding mild surges in COVID-19 human case numbers in March and August 2022 [60]. The estimated number of unreported cases is likely to be much higher than the reported cases [61, 62]. This is also reflected by serological surveys conducted by Kolawole, Tomori [62], who found seroprevalences of the human population of the FCT of 24.2% in mid-2021. From our serological results we conclude that SARS-CoV-2 entered the human population, beyond the reach or availability of testing efforts of the public health services, and spread from there in the animal population. Individuals from all species tested within our study framework were affected.

However, there are notable differences in the level of seropositivity between the different species. Whereas in the dry season seropositivity in ruminants was similarly low in all three species, in the rainy season the rate increased 6-fold in cattle compared to 12-fold in goats, suggesting a difference in intensity of exposure between the two species. This can be explained by colder and more humid weather, less ultraviolet radiation, and the increasing densities during this season [63, 64]. This is especially the case for the small ruminants which are kept dry in roofed, thus dark shadowed areas where people also seek shelter from the rains. These spaces are limited in the abattoir, which is why the ruminants and people are coming closely together for the rainy periods. Even though we were not able to conclusively identify viral RNA in any of the animals, remarkably, the only samples with questionable qPCR results were of two goats sampled in rainy season, which may support the hypothesis of increased transmission earlier in rainy season.

The low number of the wild small mammals’ samples does not allow statistical analysis, but remarkably, three out of six shrew sera tested positive in the dry season compared to none in rainy season. *Crocidura fulvastra*, belonging to *Crocidura olivieri* complex [54], are common commensals of humans, generally living solitary but in close proximity to other individuals, with which they communicate with loud shrieks [65]. Activity is almost completely nocturnal, with peaks just before dawn. During the day, they hide in burrows like found at the side of the river bed. With a high metabolic rate relying on the constant intake of protein sourced from insects, small vertebrates or carrion [66], the scavenging shrews probably probe into the human-dominated spaces to feed on their remnants deeper in dry season than in rainy season, when access to food such as maggots is easy due to dense abundance in the abattoir. Of the three individuals of *Rattus norvegicus* tested in the ELISA, one had antibodies against SARS-CoV-2. It is found worldwide as a commensal in human settlements and is known to be susceptible to SARS-CoV-2 [41].

### Human niche construction and critical (global) connectivities in the abattoir

The observed examples show uncountable daily transmission options resulting from the intense multi-species entanglement [67–69]. Exchanges driven by human behaviour, such as the slaughtering practices, keeping animals in the abattoir or caring for sick animals, are intensified through a compressed, shared and central place [6]. This challenges an anthropocentric notion existing in current literature of putting blame on certain other species as pathogenic “villains” or “disease reservoirs”, as life is lived in a multispecies community in the abattoir, constituting a wide variety of biological features, such as morbidity and immunotolerance. In this community each individual strives to survive in continuous re-negotiation with other species in a changing environment [70, 71]. Moreover, this engagement of humans and other species is not only limited to the abattoir but extends to the surrounding settlement and beyond [72–75].

Against this backdrop, the embedding of the abattoir within the settlement makes it a crucial site for investigating zoonotic pathogen circulation and emergence in peri-urban communities. The abattoir ecosystem is interconnected not only within the settlement, but also with neighbouring settlements, metropolitan areas, rural hinterlands, and relatively untouched wilderness. The results we present in this dense peri-urban setting show how the infrastructure of the agribusiness can create kinds of ‘critical connectivities’ that enable pathogen transfer by movements of all kinds of individuals from the ‘wild’ to the peri-urban– from non-human dominated landscapes across rural farmlands and settlements to feedlots in lairages, slaughterhouses, rivers, with human and non-human species and products into the urbanized settlements [76] and vice versa. The human-made and human-dominated environments are taken up and utilised by other species, blurring into each other with no clear boundaries and having their own porous “virosphere” that challenges presumed human control through multi-species entanglements [5]. Human behaviour and the constant, extensive inflow and outflow of goods and animals are driving the modification of the abattoir as a niche [77]. However, humans are continuously joined in this niche construction by the behaviours of domestic and peri-domestic animals as well as an increase in synanthropic wildlife and zoonotic host diversity, all of which together influence host-pathogen-ecologies and disease dynamics [78–80]. We believe a study like this conducted at an abattoir setting may be critical to the understanding of emergence of new potential pandemic viruses.

And yet, interestingly, investigations of similarly interwoven multi-species environments, for example in Thailand [81] and China [82], revealed that communities in close physical proximity to bat caves and with anthropogenic driven contact of humans and bats have a significantly higher SARS-related coronavirus seroprevalence than elsewhere. In our study, we have not been able to research this ambiguous protective aspect of such environments due to the lack of human serological investigations. But further research could follow this thinking on how the realities and multifaceted arrangements of volatile places like this abattoir are reflected in the global understanding of and preparedness against zoonotic disease emergence.

Global urbanization creates diverse spaces, from highly technoscientific city centres in the Global North, where biosecurity measures in the sense of compartmentalization are perfected, to areas of high development and growth, where processes of destruction and construction, and the encroachment to more “natural ecosystems” that are less modified by humans are most prevalent (Brenner and Ghosh 2022). In these hinterlands, human settlements blur with what is considered wilderness, creating a dynamic space where the pursuit of survival by humans, domestic animals and wildlife intersects. This dynamic space is often implicated in the spillover and emergence of novel pathogens, but there is a tendency to overlook the global circuits of capitalism that lead to the externalization of production, implying further encroachment, land grabbing, and deforestation, that contribute significantly to these issues [83]. The One Health framework, with its holistic and interdisciplinary approach, attempts to consider these complex and global entanglements and highlights conservation and land management as ecological countermeasures to spillover for and with affected communities [84].

### The localities of the pandemic and SARS-CoV-2 in the abattoir

In the early months of the COVID-19 pandemic, the world did not know how it would unfold in Africa [85]. There was much fear about the devastating impact on the weak health infrastructures of many African countries and economic limitations of people and governments alike [86]. Nonetheless, our fieldwork reveals frictions between global health agendas, their biosecurity concerns and priorities and the local realities [87–89]. Even though seroprevalence surveys show SARS-CoV-2 circulating in the human population in Nigeria [62], the virus itself and its disease was not a daily reality or concern for people. There are many reasons for this, one of which is that many other infectious diseases of serious public health concern occupy the health infrastructure and the health-seeking behaviours of the population [20].

Serological findings indicate exposure of the animal study population to SARS-CoV-2, suggesting virus circulation among the human population and introduction by them. This emphasizes that infectious pathogens can persist undetected in communities, not being seen and felt in their daily lives, as reports from our study participants highlighted. This is especially the case when symptoms are similar to other known diseases, when health and diagnostic capacities are limited and when the affected population is primarily at the economic margins of society. Additionally, we found an active medical pluralism among butchers, animal owners, and market sellers both for themselves and for their animals. Access to a variety of resources, including herbal healers, pharmacies and para-pharmaceutical vendors, veterinarians, and a health clinic in and around the abattoir, allows individuals to choose health care and treatments ranging from herbal, local or all-purpose-medicine to biomedical interventions like painkillers, vitamins or (unprescribed) antibiotics [90, 91]. These choices are based on financial means, knowledge and perception about certain diseases, their symptoms and the available medications, often resulting in people not seeking or receiving clinical medical attention. During the most restrictive months of the pandemic, testing was not readily available in health care facilities and was too expensive for the majority of the population.

Furthermore, our findings highlight the challenges faced by abattoir workers during the pandemic, echoing existing research on its impact in Africa. National restrictions on social activities significantly affected Nigeria’s informal sector, a vital component of the economy and the main livelihood source for many in the abattoir [92]. Informal workers, lacking job security, pensions, insurance, and healthcare coverage, were disproportionately affected by these restrictions, relying heavily on daily income for survival [93, 94]. Shutdowns of businesses, markets, religious centres, and schools, along with restrictions on public gatherings, were implemented to curb virus transmission [95]. Compliance with social distancing directives was hindered by factors like economic necessity, ignorance and defiance [96]. Our findings underscore the persistent need for daily income in an environment where multiple diseases are endemic.

## Conclusion

The unique entanglement of the abattoir in dense surrounding settlement with all its multi-species population, their labour, health seeking behaviours and intense influx and efflux makes it a perfect site for inter-species transmission of pathogens, as is the case in countless places around the world and particularly in the global South. Due to the lack of regulation, these places are a blind spot in health surveillance, pandemic preparedness measures and response mechanisms [97].

Urbanization does not stop at the borders of metropolitan areas, but is accompanied by an ever-increasing demand and production of animal protein for human consumption in Nigeria and elsewhere (FAO 2019; Brenner and Gosh 2022). This industry, pushed to the margins of urban centres, leads to the critical connectivities described above and, as we show, its webs extend from wildlands over rural areas to bustling cities. The approach we took, by embracing ethnographic data and not just looking at biological samples and blunt statistics, brings out details of situated complexity that are invaluable in the development of sustainable mitigation steps to enhance One Health and outbreak prevention. In the absence of human and environmental samples, our conclusions regarding the complex patterns of pathogen circulation warrant further investigation through expanded sampling strategies that include both human and environmental sources within the abattoir, along with extended longitudinal data collection beyond a single year to assess seasonal variations in SARS-CoV-2 prevalence.

Nevertheless, mobilising the presented veterinary and ethnographic insights is an attempt to fill the research gap on SARS-CoV-2 exposure of animals on the African continent. Though without laboratory data on past and present SARS-CoV-2 circulation in humans, we highlighted here that humans are just one entangled actor among many other species– domesticated, wild or somewhere in-between– in this abattoir setting and potentially in many others in Nigeria and beyond that need a deeper interdisciplinary focus under a One Health framework. The inherently close contact between humans and animals and the potential exchange of different pathogens paired with the critical interconnectivities driven by urbanization, potentially make these places perfect breeding grounds for zoonotic pathogens. However, coming from a holistic approach in a One Health perspective, the interdisciplinary exploration and thus, granular knowledge of such multispecies live worlds, their sensitivity, but also their potential resilience, and creative responses to changes are crucial to develop well-tailored interventions.

## Electronic supplementary material

Below is the link to the electronic supplementary material.

Supplementary Material 1

Supplementary Material 2

Supplementary Material 3

Supplementary Material 4

Supplementary Material 5

## Data Availability

The laboratory datasets supporting the conclusions of this article are included within the article and its supplementary files 1-3. Further insights into the anthropological field data can be made available upon request.

## Abbreviations

COVID-19: Coronavirus Disease 2019
FCT: Federal Capital Territory
FLI: Friedrich-Loeffler-Institute
NCDC: Nigeria Centre of Disease Control and Prevention
NVRI: National Veterinary Research Institute
RKI: Robert Koch-Institute
SARS-CoV-2: Severe Acute Respiratory Syndrome Coronavirus Type 2
RT-qPCR: Reverse transcription quantitative polymerase chain reaction
ELISA: Enzyme-linked immunosorbent assay
RNA: Ribonucleic acid
DNA: Deoxyribonucleic acid
PPE: Personal protective equipment
PHC: Primary healthcare centre
cq-value: Cycle of quantification value
