# Supplementary material for: Negotiating cohabitation in a Nigerian abattoir: One Health perspectives of human-animal-ecosystem interactions examined in the light of the SARS-CoV-2 pandemic
Source: One Health Outlook. 2025 Jul 16;7:37. doi: 10.1186/s42522-025-00161-9 (PMC12265145; doi:10.1186/s42522-025-00161-9)
Supplement: Supplementary file 4 — Supplementary Material 4 [file 42522_2025_161_MOESM4_ESM.docx]

**Supplement 4: Table of Quotes and Laboratory data**

| Humans’ activities at the abattoir |
| --- |
| “People use to sleep here but not in all the departments. Like me now, it is not compulsory that I must sleep here but I have to have somebody that is sleeping here. Like in the night, some bring cows in the night. […] Some people use to offload at night. And cows use to fight in the night. Somebody must be there, yes. [...] And some people, because of the condition of their villages, no employment, no steady place, they would just come and they will hang around anywhere they see themselves.”  Interview, animal dealer, dry season  “That animal they kill here, cow, sometimes camel, sometimes they bring camel here, then the ram, sheep, goat, and the goat itself that is an animal that is common in abattoir here. Actually, you know I have goat and some all these chickens, and ducks, these native ones at my home.”  Interview, butcher, dry season  “Yes, in addition to the regular ones we have in the abattoir, there are times where I come in contact with, as a vet I do some private practice, so yes I come into contact with companion animals, dogs, cats, poultry.”  Interview, veterinarian, rainy season  “Sometimes, I use traditional medicine. I practise traditional medicine. If I take tablets, they sometimes don’t work on my body, so I turn to traditional remedies. When I have a fever, for example, before taking pharmaceutical tablets, I first use traditional medicine. If the fever doesn’t go away completely with the traditional remedy, I’ll then take tablets to finish the treatment. I make sure to complete the process properly, and afterwards, I usually don’t have any more trouble with the illness.”  Interview, butcher, rainy season  (translated from Pidgin English; original quote: *“In times I do local medicine o. Me I dey do local medicine. If I do tablet now sometime it no go work for my body, I go for local medicine. […] Fever now, if fever done catch me, before I go take tablets I go take the local one. So that if I take the local one if it remains [the fever], the tablet I go take again. I go complete ‘em and make sure go finish. Then I go ahead and no have problem again with sickness.”*)  “You know at time cow use to enter different bush, night and day. Some of them use to carry spirit in their body. There are good and bad spirits and you inhale them. […] So, you are killing them every day and can breathe in the spirits, that thing can affect you at time. So that is what is happening mostly.”  Interview, animal dealer, rainy season |
| **COVID-19 - impact and perception** |
| “Before Covid pandemic they would slaughter up to 50 cows at one time in the slaughterhouse. But governmental hygiene restrictions now allow only 25 cows and a cleaning afterwards.”  Interview notes with a Mallam, dry season  “During Covid he lost too much and now he is working as an assistant butcher. The other interlocutor explains how he was lucky but still people bought less. Now the prices are also very high. Before Covid, his average cow would be 300.000 Naira, now it is double that price. He continues and says that he does not know of anyone who got sick of Covid in the abattoir: `It did not affect our health but our pockets.´”  Interview notes with a butcher and animal dealer, dry season  “It is a lie. It is a disease for countries like China, in Europe and America but not for me. Not for Nigeria. I do not see it - not like I see malaria, typhoid, tuberculosis around here. It made the government beg and begging is not good. It made business really hard during lockdown. Only two market days were allowed then. I know no one who had Covid or know anyone who knows anyone who had it.”  Interview notes with an animal dealer, dry season |

| Ruminants’ activities at the abattoir | | | | | | |
| --- | --- | --- | --- | --- | --- | --- |
| “At times we buy female ones, at times we buy bigger size, at time we buy small size. At times, like the dry season, we buy the one we can graze, that means to feed them, to keep them aside and feed them […] So I give them steady food day and night and water also steady day and night [in the lairage of the abattoir]. When I do it for 60 days I get some profit. I do it two times in the dry season.”  Interview, animal dealer, rainy season  “Yes, we do for fattening and some of them we just decided to, you know, keep them. Just I feel like keeping this one. We just keep them [in the lairage] and we keep some for fattening. […] So that is why in the process you see some of them giving birth here [in the lairage]. […] I keep them like three, four months or five months, the highest six months here.”  Interview, animal dealer, rainy season | | | | | | |
| **Laboratory results of ruminants sampled** | | | | | | |
| season | species | individuals sampled | swabs positive in IP-4 qPCR | tissue positive in IP-4 qPCR | sera* positive in ELISA  absolute numbers and percentage [Clopper-Pearson 95% CI in brackets] | |
| dry | cattle | 57 | 0 | 0 | 2/57 | 3.5% [0.4 – 12.1] |
|  | goat | 94 | 0 | 0 | 2/86 | 2.3% [0.3 – 8.2] |
|  | sheep | 5 | 0 | 0 | 0 | 0.0% |
| rainy | cattle | 120 | 0 | 0 | 22/119 | 18.5% [12.0 – 26.6] |
|  | goat | 102 | 0 | 0 | 34/100 | 34.0% [24.8 – 44.2] |
|  | sheep | 19 | 0 | 0 | 3 | 15.8% |

| Dogs’ activities at the abattoir | | | | |
| --- | --- | --- | --- | --- |
| “Some people use to keep dog here. […] some people use to go to hunt with them in dry season. So those ones use to keep the dogs because of hunting.  Interview, butcher, dry season  “Yes, dogs because we use to secure our environment [the abattoir] with dogs. We use them in the night, like around 10 pm. So, the dogs will come and roam around.”  Interview, market seller, rainy season  “Okay, so for the dogs that roam around here, most times they are usually around here [the abattoir and surrounding settlement]. It is only few owners that take their dogs home with them. And then a certain number of them you find them around in the uncompleted buildings, around here.”  Interview, veterinarian, rainy season | | | | |
| **Laboratory results of dogs sampled** | | | | |
| season | species | individuals sampled | swabs positive in qPCR | sera positive in ELISA |
| rainy | dog | 7 | no swabs taken | 4/7 |

| Rodents’ and shrews’ activities in the abattoir | | | | | | |
| --- | --- | --- | --- | --- | --- | --- |
| “Of course, many rats. They bother me at night in the quarter [in the abattoir]. Because I experience it in the quarter. Normally I see it.”  Interview, cleaner, rainy season | | | | | | |
| **Catching data and laboratory results of small mammals** | | | | | | |
| season | species | trapping rate | individuals caught | organs positive in qPCR | swabs positive in qPCR | sera* positive in ELISA |
| dry | *Rattus norvegicus* | 2.7 % | 2 | 0 | 0 | 0/1 |
|  | *Crocidura fulvastra* | 20.2 % | 15 | 0 | 0 | 3/6 |
| rainy | *Rattus norvegicus* | 2.4 % | 2 | 0 | 0 | 1/2 |
|  | *Crocidura fulvastra* | 17.1 % | 14 | 0 | 0 | 0/6 |
|  |  |  |  |  |  |  |
|  |  | *serum could not be retrieved from all animals | | | | |
