# Supplementary material for: Negotiating cohabitation in a Nigerian abattoir: One Health perspectives of human-animal-ecosystem interactions examined in the light of the SARS-CoV-2 pandemic
Source: One Health Outlook. 2025 Jul 16;7:37. doi: 10.1186/s42522-025-00161-9 (PMC12265145; doi:10.1186/s42522-025-00161-9)
Supplement: Supplementary file 5 — Supplementary Material 5 [file 42522_2025_161_MOESM5_ESM.docx]

**Collection of Semi-Structured Questionnaires**

**Questionnaire for Slaughterhouse Butchers**

Biographic background:

- For how long have you worked as a butcher?
- How did you become a butcher?
- Why do you work as a butcher?
- How does a workday look like for you in the abattoir but also afterwards?
  - Do you have a side business? What is it?

The abattoir as a multi-species house:

- Where do you live? (mental map)
- Where do you spent most of your time? (“I am here. This is our barrack.”)
- What activities do you do here in the abattoir apart from slaughter?
- I see the place is changing a lot over the day – from slaughter in the morning to the evenings. How do you experience it?
- Do people also sleep here in the abattoir? Where?
- When you rest or eat in the abattoir, where do you do it and why?

Function of blood:

- I see a lot of blood here every day. How do you feel about it?
- Is blood simply a by-product of the slaughtering process for you or can it have another function?
- How do you protect yourself against the cows?
- Why is blood important for charms?
  - Where do these charms come from?
- I also observed once how butchers put their hands into the blood of the throat cut of a bull that put up a big fight. Or that they put their fist into the hot stomach of a cow.
  - Do you know why they did/do that?
  - Does your view of a fighting cows changes compared to the ones that are not resisting much?
  - Do you make a difference if it is a male or female cow (cow/bull)?
- Could you tell me more about the local wrestling / fighting?
- I also see people eating in the abattoir, sometimes some also snack bits of the meat they cut off.
  - Do you know why they do it?
  - What parts are these specifically?

Health seeking / health challenges:

- What do you do if you get injured?
- How is it different when you get injured by the cow horns?
- Do butchers suffer from specific health challenges?
  - I If yes, can you tell me more about it?
  - Do you have any?
  - What do you do about it?
  - Where does it come from?

Killing ontologies:

- Could you tell me more about the hunting practice that people here do?
- Do they use the dogs around the abattoir for it? Are they specially trained?
- How do they care for them?
- Do you know when people go hunting with their dogs here?
- Where do they go to?
- How often do they go?
- What influence have the dry and rainy season on it?
- What kind of differences are there when killing a cow in the slaughter house or when killing a hunted animal? (halal?)
- Where do you/they sell the animals?

**Questionnaire for Animal Sellers and Dealers**

Biographic background:

- How long have you been in business?
- How many animals do you normally own?
- Where do you get the animals from? How do you get them here?
- How many do you sell per day / week?

The abattoir as a multi-species house:

- Where do you live? (mental map)
- Where do you spent most of your time? (“I am here. This is our barrack.”)
- What activities do you do here in the abattoir apart from slaughter?
- I see the place is changing a lot over the day – from slaughter in the morning to the evenings. How do you experience it?
- Do people also sleep here in the abattoir? Where?
- When you rest or eat in the abattoir, where do you do it and why?

Animal and human spaces

- How is the organization of this place for different activities (e.g., meeting people, eating, resting, feeding, etc.)?
- Can you mention the most important places for social life / work life in this environment?
- Where do you keep your animals? Are they separated from other animals?
- Do you have pets’ other domestic animals? Where do you keep them?
- What kind of (unwanted) animals do you see or which you know of that they are here, but which are not for slaughter? How do they affect you?

Environment:

- Changes of this environment and around this setting in the last 10 years?
  - How does this affect you and your animals?
- Did you experience extreme weather conditions in the last years (extreme heat or rain, longer seasons).
  - Do you notice changes of seasons or weather patterns in the last years?
  - How does this affect you and the animals?
- What do you know about climate change (Do you know climate change)?
  - How do you think it is affecting you and animals around you?
- How do the different seasons affect you, your daily life, and your means of making a living?
- Do you notice different behaviors of animals in different weather?
- How do you keep this environment clean?
  - And when do you clean?
  - What types of waste do you have?
  - Where are your water sources?
  - Where do you dispose your waste?
  - What do you do with animal waste?
- How do you think this place will change in the future?
- How is the security situation in the country affecting you and your business?

Perception of socio-cultural value of keeping/selling animals

- Do different animals have different value in society?
  - When do you sell/buy or slaughter what kind of animals?
- Do the animals you have hold specific cultural values for you or for people in the lairage (e.g., Fulani)?
- Is there a religious guideline of how animals should be treated and slaughtered?
- Are there religious taboos on how to treat or slaughter animals?
- Is there a festival/religious practices in the market (or in your area of the market) that brings people together – praying, sharing food, etc.?

Social structure

- How do you structure the social life here? Are there hierarchies?
- What different groups are there (ethnic but also organizational, e.g. unions)? Who is talking to / reporting to / collaborating with whom?

Network connections

- Could you tell us more about the products that come into this place?
  - From where
- And what products leave this place?
  - To where?
- What kind of different people come into this place?
  - From where / to where?

Participation in One Health

- Do you have a reporting system for animal and human diseases?
  - If yes, how does it work?
- What do you do with dead or dying animals (domestic and wild animals around?)

**Questionnaire for Veterinarians**

Background (for the ones that are new)

- For how long have you worked in this abattoir?
- How does a regular workday looks like for you?
- What are challenges and what are things you like about your work?

The abattoir as a multi-species house:

- If you had to describe the abattoir to someone who has not been here, how would you describe it to this person?
- What would you say if you were asked what this place means to you?
- Do people sleep here at night? Where?
- Why do they sleep here?
- How is the abattoir changing from the early morning into the night?
- Can you tell me more about the abattoir after main slaughter is over? What activities are done here? How is the atmosphere? Where do people mingle?
- How do you find the abattoir as a workplace?
- Could you tell me more about the dogs that roam around the abattoir?
  - Who owns them?
  - For what purposes?
  - Do the owners have a close connection towards the dogs?
  - Who is going hunting and when?
  - How are they treated when sick?
  - Are they regularly treated for common health risks?
  - Are they regularly dewormed?

Social structure

- How do you structure the social life here? What hierarchies exist here?
- What different groups are there (ethnic but also organizational, e.g. unions)? Who is talking to / reporting to / collaborating with whom?

(One) Health & Health seeking

- How do you think the abattoir is generally affecting people’s health? Why?
- What health issues do you know of that people in this abattoir have?
  - Are there specific ones for the different professions (animal dealers, butchers, roasters, carriers, market sellers, etc)
- Could you tell me more about the Rheumatism that butchers suffer from?
  - Who made the diagnosis?
  - What do the butchers do about it?
  - Do others also suffer from it, for example the women that carry the meat etc?
- I see many medicine sellers in the market and around the abattoir every day. What do they sell and what is it for?
